# Recombinant pseudorabies virus expressing the consensus VP2 protein of porcine parvovirus 1 (PPV1) protects pigs against pseudorabies virus and PPV1

**DOI:** 10.1186/s13567-025-01592-y

**Published:** 2025-08-05

**Authors:** Xiaoxiao Tian, Haojie Wang, Hao Song, Ziyi Wei, Xulong Zhu, Guoqing Liu, Mingxia Sun, Xinyi Huang, Meng Chen, Yandong Tang, Haiwei Wang, Yongbo Yang, Tongqing An

**Affiliations:** 1 State Key Laboratory for Animal Disease Control and Prevention, Harbin Veterinary Research Institute, Chinese Academy of Agricultural Sciences, Harbin, 150069 China; 2 Heilongjiang Veterinary Biopharmaceutical Engineering Technology Research Center, Harbin, 150069 China; 3 Heilongjiang Provincial Key Laboratory of Veterinary Immunology, Harbin, 150069 China

**Keywords:** PRV variant, PPV1, consensus VP2, genetic stability, chimeric viral vector vaccine

## Abstract

Pseudorabies virus (PRV) and porcine parvovirus type 1 (PPV1) are major pathogens that cause reproductive disorders in sows, and mixed infections are frequently detected on pig farms. In this study, a recombinant PRV (rPRV-VP2) based on a PRV variant (HLJ8) was constructed to express the PPV1 VP2 protein. To ensure safety, four virulence genes (TK, gE, gI, and UL39) of PRV HLJ8 were deleted, followed by further evaluation of safety and immunogenicity in piglets. The PPV1 VP2 expression cassette was inserted into the gE/gI deletion site, enabling successful expression of the VP2 protein, which exhibited hemagglutination activity with a titre of 1:64. Animal experiments demonstrated that rPRV-VP2 elicited robust antibody responses against both PRV and PPV1, providing complete protection in both mice and piglets. Compared with commercial PRV and PPV1 vaccines, rPRV-VP2 conferred comparable or superior protection, resulting in not only no clinical symptoms but also significantly reduced viral loads in tissues. The deletion of the gE gene can distinguish wild-type virus infection from vaccine immunity in clinical applications. Overall, rPRV-VP2 offers a safe and effective strategy for dual protection against PRV and PPV1, reducing vaccination costs in pig production.

**Supplementary Information:**

The online version contains supplementary material available at 10.1186/s13567-025-01592-y.

## Introduction

Pseudorabies (PR), also known as Aujeszky’s disease, is a notifiable viral disease that causes significant economic losses in the swine industry. Infected pigs display a broad spectrum of clinical symptoms, including neurological and reproductive disorders, fever, dysphagia, and weakness, with high morbidity and mortality, particularly in piglets [1]. The causative agent, pseudorabies virus (PRV), belongs to the subfamily *Alphaherpesvirinae* within the *Herpesviridae* family [2]. It is a double-stranded DNA virus with a genome of approximately 150 kb, encoding more than 70 viral proteins [3]. Some are essential for viral replication, such as gB, gD, gH, gL, and gK, whereas others are virulence factors, such as TK, gE, and gI [4, 5]. In China, the first report of a PRV outbreak occurred in 1947, and the Bartha-K61 vaccine was imported from Hungary to China in 1979 [6]. From the 1990s until late 2011, over 80% of pigs in China were vaccinated with the Bartha-K61 vaccine, effectively controlling PR. However, beginning in late 2011, PR occurred on many large pig farms that have been vaccinated with the Bartha-K61 vaccine [7–9]. Further studies have demonstrated that the Bartha-K61 vaccine does not provide complete protection against PRV variants [9, 10]. As a result, the development of vaccines targeting PRV variants has attracted widespread attention.

Porcine parvovirus (PPV) is a small, nonenveloped virus that is classified into eight genotypes, including classical PPV type 1 (PPV1) and seven novel types (PPV2-PPV8) [11]. Among the eight types, PPV1 is the most prevalent and is considered one of the major pathogens causing reproductive disorders in sows with stillbirth, mummification, embryonic death, and infertility [12]. In recent years, PPV1 variant strains have been reported successively [13]. The genetic diversity and high mutation rate (10^–4^–10^–5^ mutations/nucleotide/year) have led to the emergence of increasing numbers of new PPV1 variants, and possible antigenic variation may influence effective vaccination against PPV1 [14, 15]. Both PPV1 and PRV can cause reproductive disorders in sows and are often found in coinfections. The development of a PRV-PPV1 recombinant vaccine could achieve effective protection against both diseases with a single immunization. The PRV genome is large and contains numerous nonessential genes that can be used for foreign gene insertion, making it an ideal vector vaccine. To date, PRV has been widely used as a vector to express other pathogens, including viruses (classical swine fever virus [16–18], porcine circovirus type 2 [19, 20], porcine reproductive and respiratory syndrome virus [21, 22], and PPV1 [23, 24]), parasites [25, 26], and bacteria [27]. Although most recombinant viruses are highly safe and can induce animals to produce specific antibodies that provide complete protection, some, such as PRV SA215/VP2, offer only partial protection [23]. To improve the immune response, a novel rPRV (rPRV HB98-VP2-IL6) was constructed to co-express IL-6 and VP2. Although, compared with rPRV-VP2-EGFP, rPRV-VP2-IL6 exhibited superior immune protection in mice, complete protection was not achieved [24]. Given that PRV and PPV1 are major pathogens that cause reproductive disorders in sows and that the existing PRV and PPV1 recombinant vaccines do not provide complete immune protection, this study will optimize and improve upon current research to construct a recombinant vaccine for PRV and PPV1 that offers safety, robust immunogenicity, and complete protection.

To ensure the safety of the vaccine, a PRV variant (HLJ8) with deletions of the TK, gE, gI, and UL39 virulence genes was constructed. Furthermore, all the full-length PPV1 VP2 gene sequences (*n* = 143) in China were downloaded from GenBank, and their consensus sequence was obtained by sequence alignment. Then, codon optimization was performed with reference to pig codon preferences, and the Kozak sequence was added at the 5' terminus to improve the expression of the VP2 protein. A recombinant PRV expressing the consensus PPV1 VP2 protein was subsequently constructed, and its immune response in mice and piglets was assessed. The results demonstrated that the recombinant virus effectively induced the production of specific antibodies against the PRV gB and PPV1 VP2 proteins in both mice and piglets, providing complete protection.

## Materials and methods

### Viruses and cells

The PRV HLJ8 strain (GenBank accession no. KT824771.1) was isolated from the brain tissue of piglets in Heilongjiang Province in 2013 by our laboratory [28]. PRV-ΔUL39 is a virus with deletion of the UL39 gene (a virulence-related gene) of PRV HLJ8, which was generated and stored in our laboratory. PRV and PPV1 were propagated in Vero cells and PK-15 cells, respectively. Vero, BHK-21 and PK-15 cells were grown in Dulbecco’s modified Eagle’s medium (DMEM, USA) supplemented with 10% foetal bovine serum (Gibco) at 37 ℃ with 5% CO_2_.

### Construction of the pCA-arm-eGFP transfer vector

To generate a recombinant PRV lacking the virulence-related genes gE and gI, a transfer vector, pCA-arm-eGFP, was constructed according to previous research [29]. First, the left and right homologous arms were amplified from the PRV HLJ8 genome by PCR using primer pairs left arm-F/R and right arm-F/R, and the left homologous arm was subsequently cloned and inserted into the *Sal* I (R0138S, NEB)-linearized pCAGGS plasmid to construct the pCAGGS-L-arm. The eGFP gene was amplified from the pCA-eGFP plasmid by PCR using primer pair pCA eGFP-F/eGFP-R, after which the donor template (eGFP-R-arm) was obtained by fusion PCR. The pCAGGS-L-arm plasmid was linearized using *EcoR* I and *Nhe* I and subsequently purified along with the PCR product eGFP-R-arm. Finally, the recombinant transfer plasmid (pCA-arm-eGFP) was constructed by a Monad kit (MC40201M) with the eGFP-R-arm and linearized pCAGGS-L-arm.

### Consensus sequence of the PPV1 VP2 gene

All full-length PPV1 VP2 gene sequences (*n* = 143) in China were downloaded from GenBank, and the consensus sequence was obtained by sequence alignment using MAFFT software. Codon optimization was performed on the basis of pig codon preferences, with a Kozak sequence added at the 5' terminus to increase the expression of the VP2 protein. Additionally, homologous arms were introduced upstream of the promoter and downstream of the VP2 gene to facilitate recombination between the VP2 expression cassette and the PRV genome. The optimized VP2 gene expression cassettes, including the homologous arms, were subsequently sent to BGI Technology (Beijing Liuhe) Co., Ltd., for synthesis and were subsequently cloned and inserted into the pMV vector, resulting in the construction of the pMV-arm-VP2 transfer vector.

### Plaque assays

For plaque purification, Vero cells cultured in 6-well plates were inoculated with serial dilutions of virus supernatants at 37 °C for 2 h. Following the infection period, the cells were washed with PBS and then overlaid with a mixture of 2% low melting point agarose and 2 × DMEM containing 4% fetal bovine serum. The cells were incubated at 37 °C in 5% CO_2_. Individual plaques were subsequently selected and propagated in 12-well plates seeded with Vero cells and further identified by PCR. Multiple rounds of plaque purification were carried out until a single target virus was obtained. For plaque staining, the same steps described above were used, but instead of individual plaques being selected, the cells were fixed with 4% paraformaldehyde for 30 min. After being washed three times with PBS, the staining solution (saturated crystal violet) was added and incubated for 15 min. The staining solution was then aspirated, followed by three washes with PBS until the background was clear. The cells were air-dried before being observed.

### Construction of the recombinant PRV

The genomic DNA of PRV was extracted following a previously described method [30]. The sgRNAs targeting the gE, gI and TK genes were stored in our laboratory (Additional file 1), while the sgRNAs targeting the eGFP gene were constructed in this study as previously described [31]. First, BHK-21 cells were co-transfected with 2 μg of pX330-sgRNA TK1 and pX330-sgRNA TK2 using PEI transfection reagent and then inoculated with PRV-△UL39 at 0.1 multiplication of infection (MOI). The supernatants were collected at 60 h post-infection (hpi) for plaque purification. Several plaques were randomly selected and propagated in Vero cells. Viral DNA was then extracted using the EasyPure Viral DNA/RNA Kit (TransGen) according to the manufacturer’s instructions and further identified by PCR using primers TK-F1/R1. Finally, a virus with the TK gene deleted was obtained. The gE and gI genes of PRV-ΔUL39/TK were subsequently knocked out using the same method.

The recombinant rPRV-eGFP was constructed using the method described above. In brief, 2.5 μg of the PRV-ΔUL39/TK genomic DNA, 0.5 μg of each of two sgRNAs targeting the gI and gE genes, and 0.5 μg of the transfer plasmid pCA-arm-eGFP were co-transfected into BHK-21 cells to generate the recombinant rPRV-eGFP. After six rounds of plaque purification, a single recombinant rPRV-eGFP virus was successfully selected. The rPRV-eGFP genome was subsequently extracted and co-transfected with pCA-arm-VP2 and sgRNAs targeting the eGFP gene into BHK-21 cells. After five rounds of purification, plaques lacking green fluorescence were selected. Finally, recombinant rPRV-VP2 was obtained.

### Growth kinetics analysis

To analyse the growth properties of the recombinant viruses, Vero cells were cultured in 12-well plates and infected with PRV HLJ8, PRV-ΔUL39/TK/gE/gI, or rPRV-VP2 at an MOI of 0.1. At 1 hpi, the cells were washed three times, and 1 mL of fresh 2% FBS DMEM was added to each well. The cells were then incubated at 37 ℃ with 5% CO_2_. The cultures were harvested at 0, 4, 8, 12, 24, 36, 48 and 60 hpi. The titres of all the samples collected at various time points were determined in triplicate using Vero cells, and the average titre was calculated as described previously [29].

### Genetic stability of the recombinant virus

To evaluate genetic stability, rPRV-VP2 was serially passaged to the 20^th^ passage in Vero cells. The infected cells were monitored for the absence of green fluorescence, and the cells were collected when the cytopathic effect (CPE) reached 80%. After three freeze‒thaw cycles, DNA was extracted from infected cells at passages 5, 10, 15 and 20, and the inserted VP2 gene was amplified using PCR and sequenced (Additional file 1).

### Immunofluorescence assay (IFA)

To detect the expression of the VP2 protein, Vero cells were infected with rPRV-VP2 and PRV HLJ8 at an MOI of 0.01. The infected cells were fixed at 24 hpi with cold absolute ethanol for 30 min, incubated with a mouse anti-VP2 monoclonal antibody (1B7), which was kindly provided by Dr. Fang Fu at Harbin Veterinary Research Institute, at 37 ℃ for 1 h, and washed three times with PBST. The cells were incubated with an anti-mouse lgG (whole molecule)-FITC antibody (1:200, Sigma‒Aldrich) at 37 ℃ for 1 h, followed by three washes with PBST. The expression of gB protein was detected by IFA as described above, using anti-gB monoclonal antibody (1E7), which was kindly provided by Dr Zhijun Tian at the Harbin Veterinary Research Institute. Fluorescence images were captured using a fluorescence microscope (Thermo Fisher Scientific).

### Western blot analysis

The expression of the PPV1 VP2 protein was further determined by western blot analysis. Vero cells were either mock-infected with DMEM or infected with PRV HLJ8 or rPRV-VP2 at an MOI of 0.1. At 24 hpi, the cells were treated with cell lysis buffer containing 1 mM phenylmethanesulfonyl fluoride. The protein samples were analysed via western blotting using anti-VP2 mouse antibody and HRP-conjugated goat anti-mouse IgG as the primary and secondary antibodies, respectively, following previously described methods [32].

### Hemagglutination and hemagglutination inhibition test

The hemagglutination activity of rPRV-VP2 was determined by a hemagglutination assay [33]. Briefly, 25 μL of PBS was added to the 96-well V-plate, 25 µL of the recombinant virus rPRV-VP2 was added to the first column and mixed well, and then 25 μL of the twofold-diluted rPRV-VP2 was added to the second column of wells, and the dilution pattern across the plate was repeated to complete the twofold serial dilutions of rPRV-VP2. A total of 25 μL of a 1% chicken erythrocyte suspension was added to the plate, which was shaken for 3 min to mix well, and the result was recorded 1 h later. The highest dilution that allowed 100% agglutination of red blood cells was the hemagglutination titre of rPRV-VP2. The hemagglutination inhibition (HI) antibody titre was determined by a hemagglutination inhibition test. In brief, 25 μL of each serum sample was serially diluted twofold, mixed with equal volumes of 4 HA units of the PPV1 strain and incubated at 37 ℃ for 1 h. Then, 25 μL of a 1% chicken erythrocyte suspension was added to the plate and incubated at 37 ℃ for 45 min. The HI antibody titre was determined when the highest serum dilution enabled complete suppression of erythrocyte agglutination.

### Safety and immunogenicity of PRV-ΔUL39/TK/gE/gI in pigs

The animal experiments were approved by the Animal Care and Ethics Committee of Harbin Veterinary Research Institute, Chinese Academy of Agricultural Sciences, China. Ten 28-day-old PRV antigen- and antibody-negative piglets were randomly divided into 2 groups. Each pig in the vaccine group was inoculated with 2 mL of PRV-△UL39/TK/gE/gI virus (1 × 10^6^ TCID_50_/mL) via intramuscular (i.m.) injection, while each pig in the control group was inoculated i.m. with 2 mL of DMEM. The clinical manifestations and rectal temperatures of the piglets were recorded daily. Blood, nasal, and rectal swabs were collected from the piglets on days 0, 7, 14 and 21 post-immunization (dpi). All the piglets were subsequently euthanized at 21 dpi; tissue samples, including the heart, liver, lungs, spleen, kidneys, tonsils, thymus, lymph nodes, brain, cerebellum, and trigeminal ganglia, were collected; and the viral genomes were detected by quantitative polymerase chain reaction (qPCR) according to a previously described method [34].

### Immunoprotective effect of rPRV-VP2 in mice

The immune response level of rPRV-VP2 in mice was also evaluated. Four-week-old female BALB/c mice were randomly divided into three groups, with six mice in each group. The mice in the immunization group were inoculated i.m. with rPRV-VP2 at doses of 10^6^ and 10^5^ TCID_50_ (200 μL/dose). The mice in the control group were inoculated i.m. with DMEM. A second immunization was performed at 21 dpi by injecting the same dose of virus or DMEM. Blood samples were collected at 0, 7, 14, 21, 28, and 35 dpi, and their body weights were recorded. All the mice were challenged i.m. with PRV HLJ8 at a dose of 1.4 × 10^3^ TCID_50_ at 35 dpi. After challenge, the clinical symptoms of the mice were observed daily, and their survival status was recorded.

### Immunological protection effect of rPRV-VP2 in pigs

For pig immunization, 35 four-week-old piglets were randomly divided into seven groups (A-G), with five piglets in each group. Groups A and E were inoculated i.m. with rPRV-VP2 (2 × 10^6^ TCID_50_). The piglets in groups B to D were vaccinated with the PRV inactivated vaccine (JS-2012-△gE/gI strain), the PRV live attenuated vaccine (TP strain), or DMEM, respectively. The piglets in groups F and G were vaccinated with the PPV1 inactivated vaccine and DMEM, respectively. A second immunization was performed at 21 dpi by injecting the same dose of virus or DMEM (except for the PRV attenuated vaccine group). At 35 dpi, groups A to D were challenged i.m. with 10^7.5^ TCID_50_ of the PRV HLJ8 strain. Groups E to G were subsequently challenged i.m. with 10^5^ TCID_50_ of the PPV1 JL-19 strain. All the piglets were subsequently euthanized at 14 days post-challenge (dpc); tissue samples, including heart, liver, lung, spleen, kidney, tonsil, thymus, lymph node, brain, cerebellum, and trigeminal ganglia, were collected; and viral genomes were detected via qPCR. A small amount of the above tissues was fixed in 4% formaldehyde, processed into paraffin sections, and stained with haematoxylin‒eosin (HE) to examine the pathological changes in the submandibular lymph nodes, lungs, tonsils, and brain tissues.

### Antibody detection of PPV1 and PRV

PRV gB and gE antibodies in mouse and pig serum samples were identified via the PRV Antibody Test Kit (IDEXX Laboratories, Inc.). PPV1 VP2 Abs in the pig serum samples were identified via the PPV1 VP2 Antibody Test Kit (Keqian Biology Co., Ltd., Wuhan), and the PPV1 VP2 antibodies in the mouse serum samples were detected using a previously described method [24].

### Statistical analysis

Data analyses were performed using GraphPad Prism 6.0 software (GraphPad Software, Inc., La Jolla, CA, USA). Statistical differences between groups were assessed using unpaired two-tailed Student’s *t* tests or two-way analysis of variance (ANOVA). A value of *p* ≤ 0.05 was considered statistically significant (* p< 0.05, ** p < 0.01, *** p <0.001, and **** p < 0.0001).

## Results

### Generation of PRV-ΔUL39/TK/gE/gI

**Figure 1:**
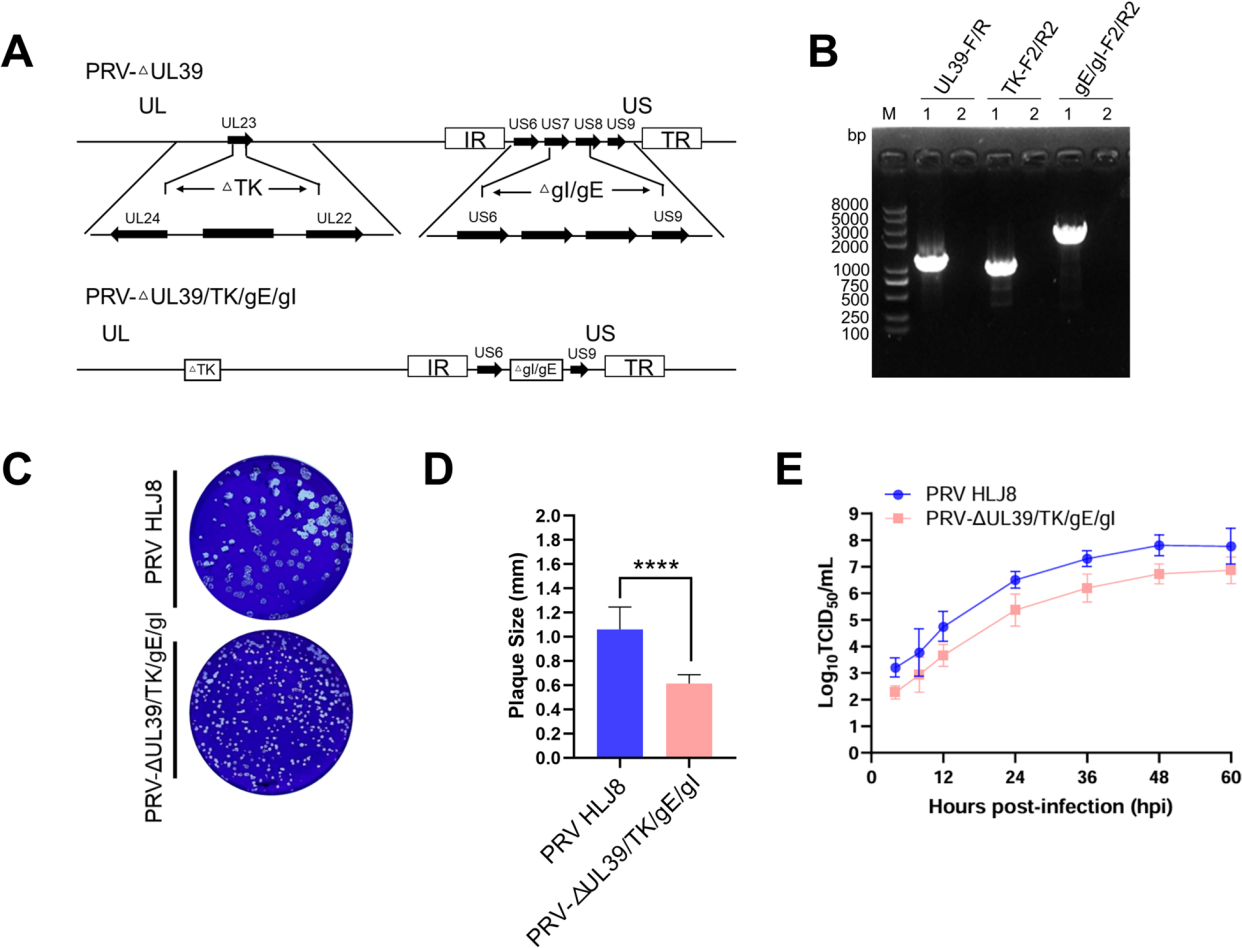
**Construction and characterization of PRV-ΔUL39/TK/gE/gI. A** Schematic diagram of the PRV-ΔUL39/TK/gE/gI construction strategy. **B** Polymerase chain reaction (PCR) identification of the deletions of the UL39, TK, gE and gI genes. Lines 1 and 2 represent PRV HLJ8 and PRV-ΔUL39/TK/gE/gI, respectively. **C** Plaque morphology of PRV HLJ8 and PRV-ΔUL39/TK/gE/gI in Vero cells. **D** Comparison of plaque sizes between PRV HLJ8 and PRV-ΔUL39/TK/gE/gI. Fifty plaques for each strain were randomly chosen to calculate plaque size. The data are presented as the mean ± SD. Statistical significance was determined using an unpaired two-tailed Student’s *t* test (****, *p* < 0.0001). **E** One-step growth curves of PRV-ΔUL39/TK/gE/gI and PRV HLJ8 in Vero cells.

The TK, gI and gE genes of PRV-ΔUL39 were sequentially knocked out by the CRISPR/Cas9 system (Figure 1A). Specifically, the Cas9 plasmids pX330-sgRNA TK1 and pX330-sgRNA TK2 were co-transfected into BHK21 cells, which were subsequently inoculated with PRV-ΔUL39 at an MOI of 0.1. The resulting virus with the TK gene deletion was purified by plaque assay and further validated by PCR using the primers TK-F1/R1 and TK-F2/R2. A virus with both UL39 and TK gene deletions was subsequently obtained, which was named PRV-ΔUL39/TK. To generate the final quadruple mutant PRV-ΔUL39/TK/gE/gI, the gE and gI genes of PRV-ΔUL39/TK were knocked out following the aforementioned process. Briefly, the Cas9 plasmids pX330-sgRNA gI and pX330-sgRNA gE were co-transfected into BHK-21 cells, which were then inoculated with PRV-ΔUL39/TK at an MOI of 0.1. The virus with deletions in gE and gI was purified via plaque assay and further verified by PCR with the primers gE/gI-F1/R1 and gE/gI-F2/R2. Finally, PRV-ΔUL39/TK/gE/gI was constructed, which was transmitted to Vero cells continuously for 20 generations and further detected by PCR using primer pairs UL39-F/R, TK-F2/R2, and gE/gI-F2/R2 (Figure 1B). Plaque experiments revealed that both PRV-ΔUL39/TK/gE/gI and PRV HLJ8 could form plaques on Vero cells, with a significant difference observed between the two (Figures. 1C and D). Furthermore, although both viruses presented similar growth curves, the propagation rate of PRV-ΔUL39/TK/gE/gI was slightly lower than that of PRV HLJ8 (Figure 1E).

### Safety and immunogenicity of PRV-ΔUL39/TK/gE/gI in pigs

**Figure 2:**
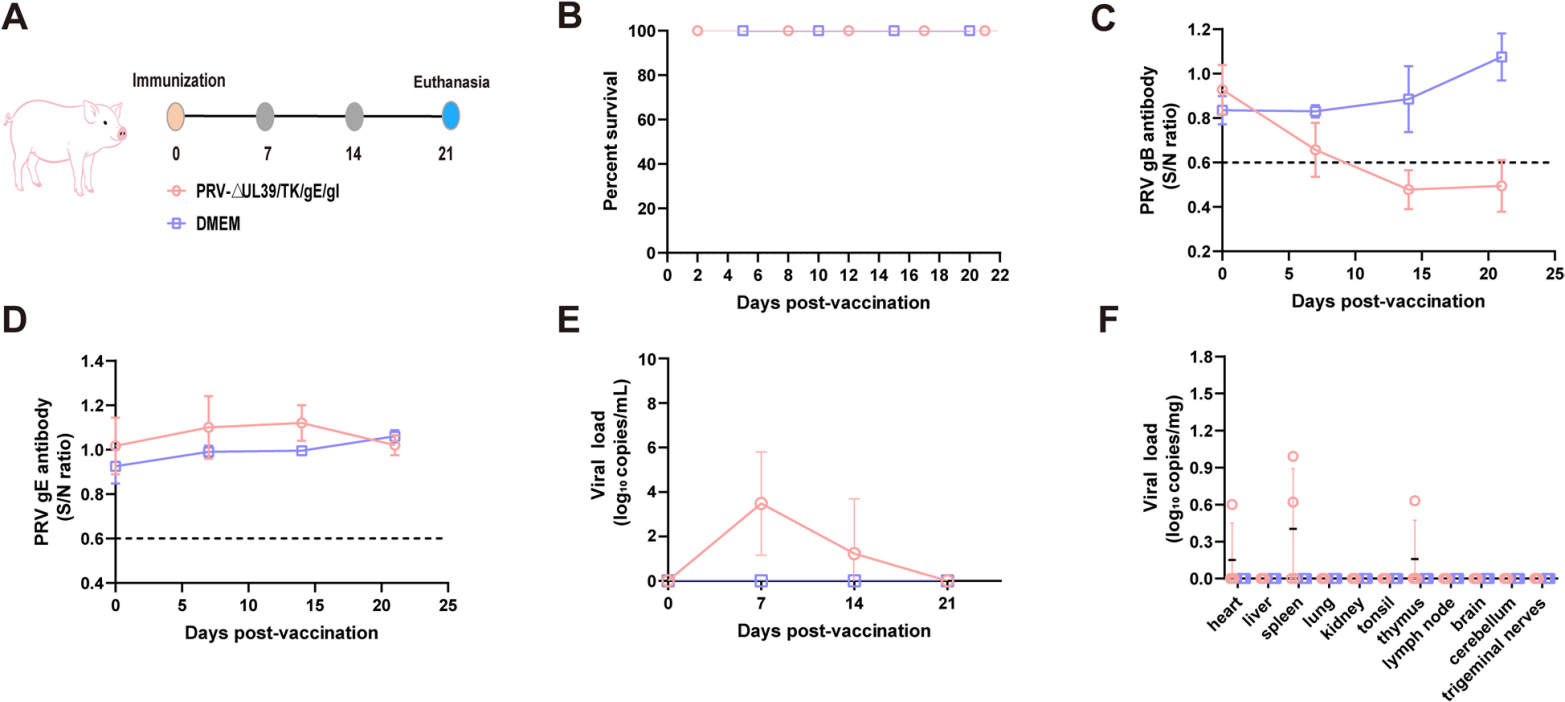
**Safety and immunogenicity evaluation of PRV-ΔUL39/TK/gE/gI in pigs. A** Schematic of the PRV-ΔUL39/TK/gE/gI immunization procedure in pigs. **B** Survival rate of pigs immunized with PRV-ΔUL39/TK/gE/gI. **C** PRV gB-specific Ab levels in pigs immunized with PRV-ΔUL39/TK/gE/gI. **D** PRV gE-specific Ab levels in pigs immunized with PRV-ΔUL39/TK/gE/gI. **E** Viral loads in serum at 0, 7, 14, and 21 dpi. **F** Viral load in tissues detected by qPCR.

The safety and immunogenicity of PRV-ΔUL39/TK/gE/gI were evaluated in pigs (Figure 2A). Following inoculation with PRV-ΔUL39/TK/gE/gI or DMEM, no clinical symptoms or mortalities were observed in the experimental pigs (Figure 2B). The PRV gB antibodies began to appear at 7 dpi and became fully positive at 14 dpi (Figure 2C). In contrast, the pigs were negative for PRV gE antibodies during the experimental period (Figure 2D). Uninfected piglets exhibited no serological reactivity to either gB or gE antigens throughout the experimental duration. The viral loads in the serum samples collected from individual piglets at 0, 7, 14, and 21 dpi were detected. Low viral loads were detected in infected pigs, with a gradual increase from 0 to 7 dpi, reaching a peak at 7 dpi, with an average level of 1.4 × 10^4^ copies/mL. The viral load subsequently progressively decreased and became undetectable by 21 dpi. No viral loads were detected in the uninfected pigs (Figure 2E). The tissue samples from surviving pigs euthanized at 21 dpi were analysed by qPCR. Low viral loads were detected in the heart, spleen and thymus, whereas other tissues tested negative for PRV (Figure 2F).

### Generation of the recombinant virus rPRV-VP2

**Figure 3:**
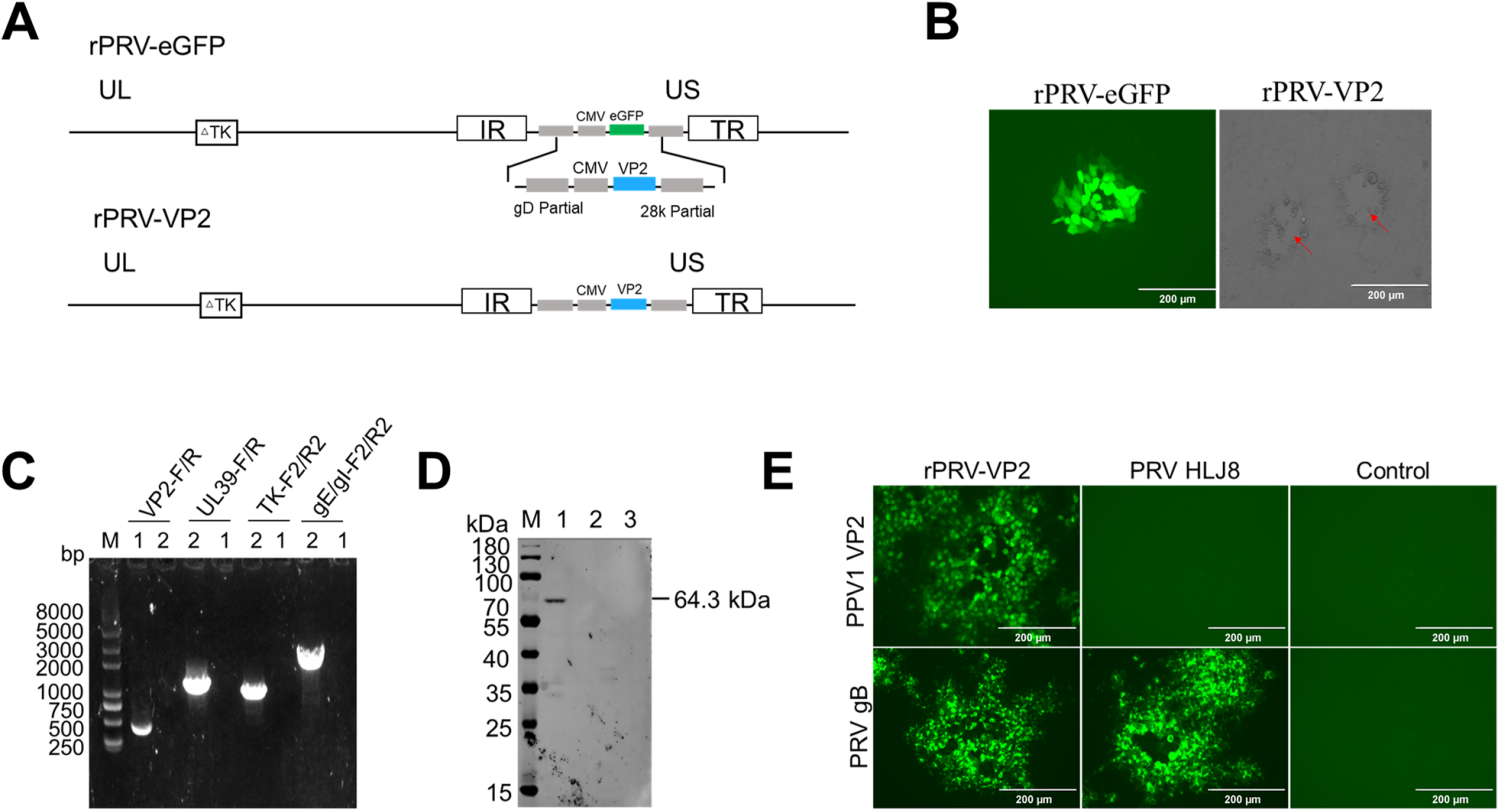
**Construction and identification of rPRV-VP2. A** Schematic diagram of the rPRV-VP2 construction strategy. **B** Plaque purification of the recombinant virus. Virus plaques are indicated with red arrows. Scale bars: 200 μm. **C** PCR identification of the insertion of the VP2 gene and deletion of virulence genes. Lines 1 and 2 represent rPRV-VP2 and PRV HLJ8, respectively. **D** Western blotting (WB) analysis of the expression of the VP2 protein in Vero cells infected with rPRV-VP2. Lines 1–3 represent rPRV-VP2-, PRV HLJ8-, and mock-infected cells, respectively. **E** Detection of the expression of the PPV1 VP2 and PRV gB proteins by an immunofluorescence assay (IFA). Scale bars: 200 μ.

The recombinant virus rPRV-VP2, which expresses the PPV1 VP2 protein, was constructed by homologous recombination via the use of eGFP as a screening marker. Initially, the intermediate rPRV-eGFP virus was generated by co-transfecting PRV-ΔUL39/TK genomic DNA, sgRNAs targeting the gI and gE genes, and the pCA-arm-eGFP plasmid into BHK-21 cells. The virus was subjected to six rounds of plaque purification (Additional file 2). To knock out the eGFP gene, seven sgRNAs were designed, among which sgRNAs 1, 3, and 7 exhibited effective knockout efficiency (Additional file 3). The rPRV-eGFP genome was subsequently extracted and co-transfected with pCA-arm-VP2 and sgRNAs targeting the eGFP gene into BHK21 cells to generate the recombinant rPRV-VP2 (Figure 3A). To obtain a pure recombinant virus lacking the eGFP gene, five rounds of plaque purification were performed until green fluorescence was no longer observed (Figure 3B). The insertion of the VP2 gene sequence into the rPRV-VP2 genome was confirmed via PCR, which further revealed the deletion of the UL39, TK, gE and gI virulence genes (Figure 3C). To evaluate the expression of the VP2 protein, Vero cells infected with rPRV-VP2 or PRV HLJ8 were lysed and subjected to western blot analysis. A specific band recognized by a mouse anti-VP2 antibody was observed in Vero cells infected with rPRV-VP2 but not in those infected with PRV HLJ8 (Figure 3D). Additionally, IFA was performed to assess VP2 protein expression in Vero cells after infection with rPRV-VP2. Positive immunofluorescence for the VP2 protein was detected in Vero cells infected with rPRV-VP2, whereas no fluorescence signal was detected in cells infected with PRV HLJ8 (Figure 3E).

### Replication dynamics and genetic stability of rPRV-VP2

**Figure 4:**
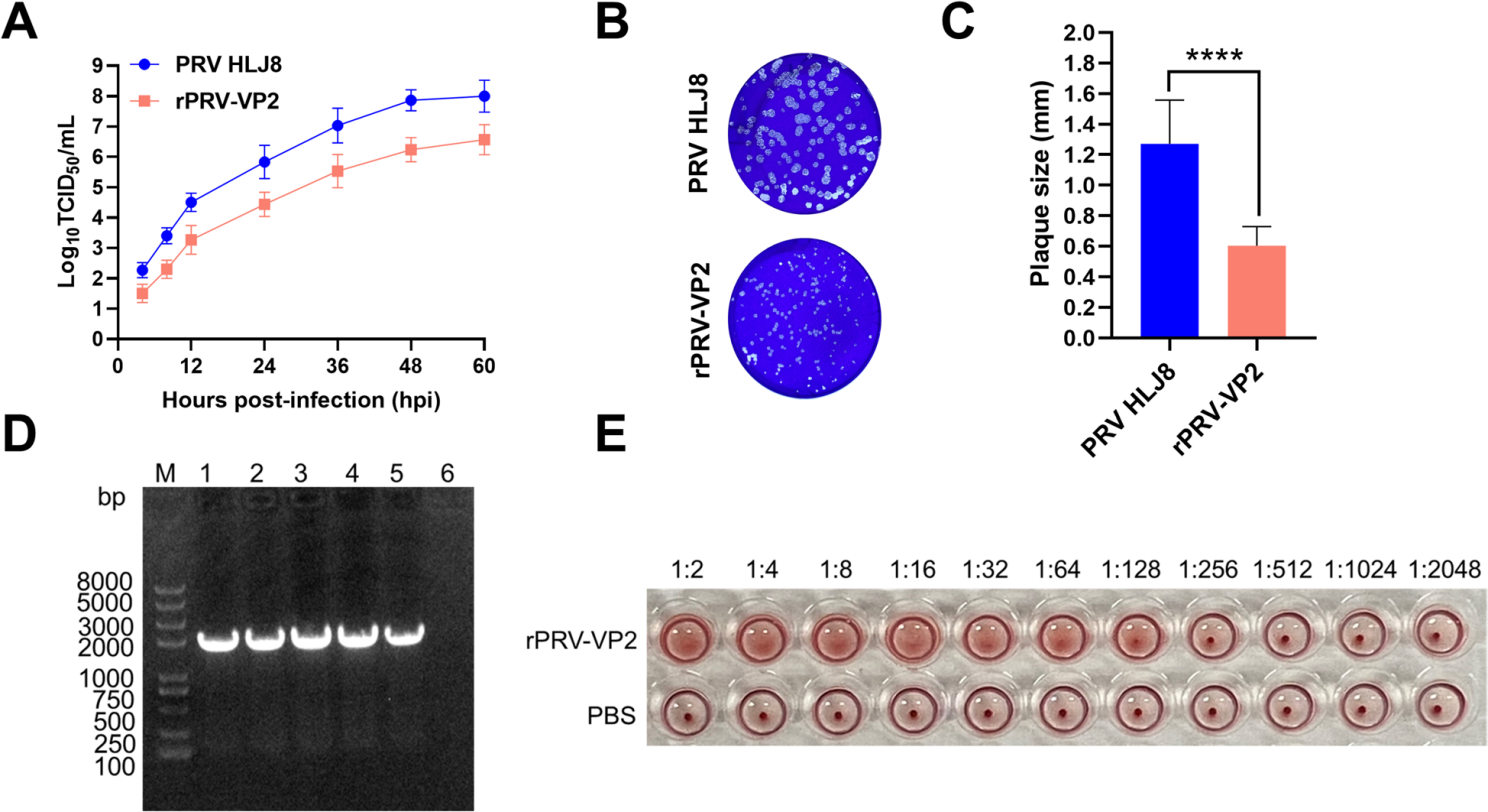
**Growth properties, genetic stability and hemagglutination activity of rPRV-VP2. A** One-step growth curves of PRV HLJ8 and rPRV-VP2 in Vero cells. **B** Plaque morphology of PRV HLJ8 and rPRV-VP2 in Vero cells. **C** Comparison of plaque sizes between PRV HLJ8 and rPRV-VP2. Fifty plaques for each strain were randomly chosen to calculate plaque size. The data are presented as the mean ± SD. Statistical significance was determined using an unpaired two-tailed Student’s *t* test (****, *p* < 0.0001). **D** PCR identification of the inserted target gene (VP2) in cell cultures from generations 1, 5, 10, 15, and 20 of rPRV-VP2. 1–5 represent the 1^st^, 5^th^, 10^th^, 15^th^, and 20^th^ passages of rPRV-VP2, respectively, whereas 6 represents the negative control (ddH_2_O). (E) Haemagglutination test of rPRV-VP2.

To assess whether the insertion and expression of foreign fragments impact the biological characteristics of the virus, one-step growth curves and plaque assays were conducted for both the rPRV-VP2 and PRV HLJ8 strains. The growth kinetics of rPRV-VP2 in Vero cells were comparable to those of the PRV HLJ8 strain, yet the virus titre was slightly lower (Figure 4A). Notably, a statistically significant difference in plaque size was detected between rPRV-VP2 and PRV HLJ8 (Figures. 4B and C). In addition, the stability of the foreign gene carried by the recombinant virus (F1-F20) was verified by PCR. The presence of an electrophoretic band at approximately 1737 bp, which was consistent with the expected results, confirmed the stable carriage of the inserted foreign gene (Figure 4D).

### Hemagglutination activity of rPRV-VP2

The hemagglutination activity of rPRV-VP2 was confirmed to be 2^6^ (1:64) (Figure 4E), indicating that the VP2 protein correctly displays the epitope required for hemagglutination activity, and the structure was similar to that of the native PPV1 VP2 protein.

### Immunological response of rPRV-VP2 in mice

**Figure 5:**
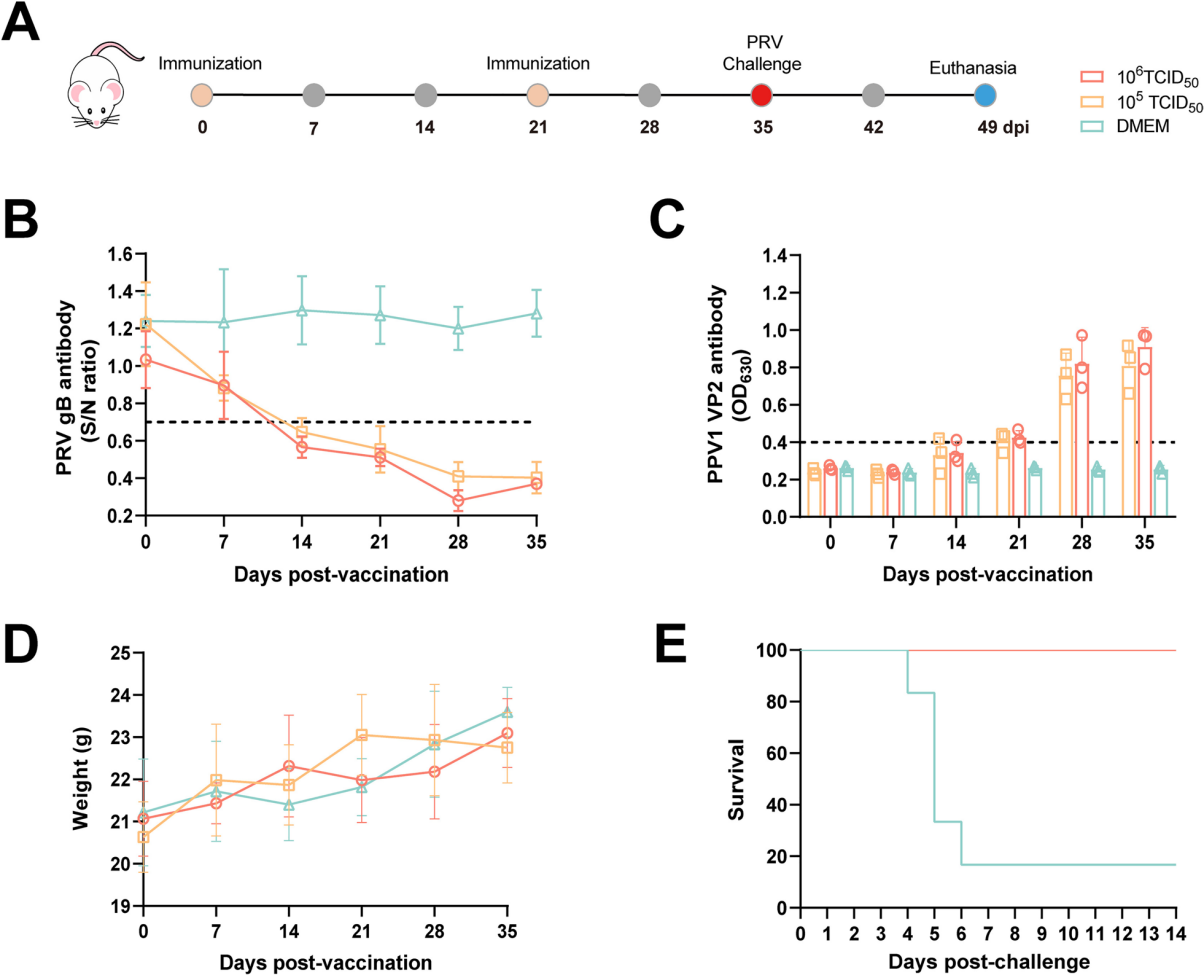
**Immunological protective effect of rPRV-VP2 in mice. A** Schematic of the rPRV-VP2 immunization procedure in mice. **B** PRV gB-specific Ab levels in mice immunized with rPRV-VP2. **C** PPV1 VP2-specific Ab levels in mice immunized with rPRV-VP2. **D** Body weight changes in mice after immunization with rPRV-VP2. **E** Survival curves of mice following challenge with PRV HLJ8.

The immunogenicity of rPRV-VP2 was initially validated in mice (Figure 5A). ELISA analysis revealed the production of anti-gB and anti-VP2 antibodies in immunized mice. The mice presented PRV gB-specific antibody titres in the serum at 14 dpi, with titres progressively increasing at 21 and 28 dpi, followed by a slight decrease at 35 dpi (Figure 5B). Similarly, the mice seroconverted to PPV1-specific antibodies starting at 14 dpi, which remained at high levels at 28 and 35 dpi (Figure 5C). Higher antibody titres were observed in the high-dose group than in the low-dose group, although the difference was not statistically significant. No specific antibodies were detected in the DMEM-treated mice. Throughout the entire immunization period, the mice presented no obvious clinical symptoms, and their body weights increased steadily (Figure 5D). Following secondary immunization, all the mice were challenged i.m. with PRV HLJ8 at 1.4 × 10^3^ TCID_50_. DMEM-treated mice displayed itching and biting behaviours, with 83.33% mortality (5/6), whereas all the rPRV-VP2-immunized mice survived (Figure 5E).

### Protection of pigs immunized with rPRV-VP2 against PRV challenge

**Figure 6:**
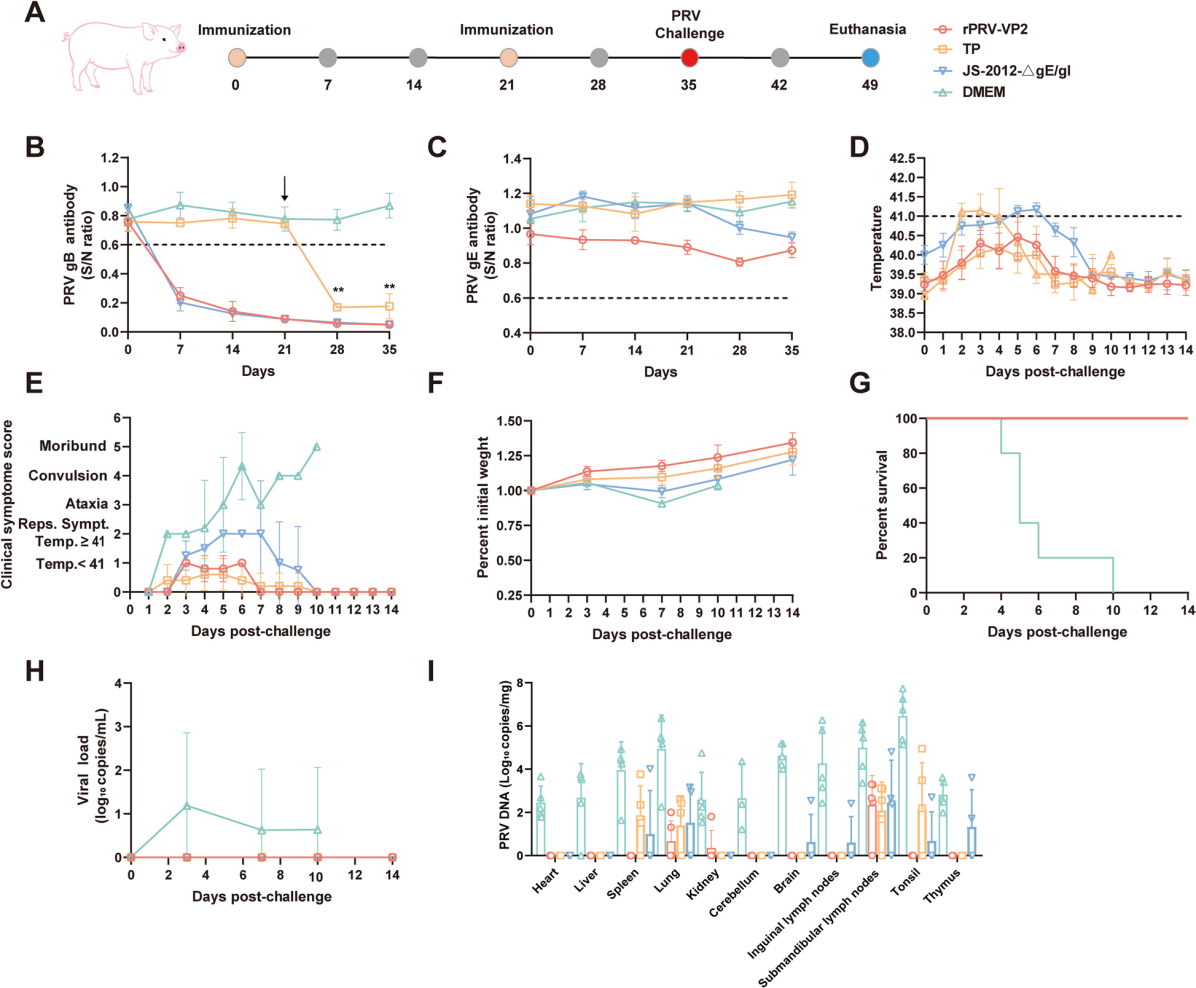
**Protection of pigs vaccinated with rPRV-VP2 against PRV challenge. A** Schematic of the rPRV-VP2 immunization procedure in pigs. TP is a commercial live attenuated vaccine, and JS-2012-△gE/gI is a commercial inactivated vaccine. **B** PRV gB-specific Ab levels in pigs immunized with rPRV-VP2. The TP group was immunized once, coinciding with the booster vaccination administered to the other groups (21 dpi, indicated by an arrow). Statistical significance between the TP group and each of the rPRV-VP2 and inactivated vaccine groups was analysed by two-way ANOVA (**, *p* < 0.01). **C** PRV gE-specific Ab levels in pigs immunized with rPRV-VP2. Rectal temperature changes (**D**), clinical symptoms (**E**), daily weight gain rate (**F**), and survival curves (**G**) of pigs following challenge with PRV HLJ8. (H) Viral load in serum at different time points post-challenge. **I** Viral load in tissues detected by qPCR.

The immunogenicity of rPRV-VP2 (Group A) in pigs was evaluated and compared with that of commercial PRV vaccines (Groups B-C) (Figure 6A). At 7 dpi, pigs in the rPRV-VP2 and PRV inactivated vaccine groups seroconverted for PRV gB-specific antibodies, with no significant difference between these two groups. At 21 dpi, booster immunizations were administered to these groups, whereas pigs in the TP attenuated vaccine group received their first immunization. One week later, although gB-specific antibodies were detected in the TP group, their levels remained significantly lower than those in the other two groups (Figure 6B). PRV gE antibodies were absent in all immunized and control pigs (Figure 6C). To evaluate the immunological protection of the vaccines, all pigs were challenged with 10^7.5^ TCID_50_ of PRV HLJ8 by intramuscular injection at 35 dpi. There were no clinical symptoms in pigs immunized with the rPRV-VP2 or PRV attenuated vaccine, whereas individual pigs immunized with the inactivated PRV vaccine presented high fever and respiratory symptoms, among which pig #20 developed severe diarrhoea at 7 dpc, nearly died at 10 dpc, and then gradually recovered. The unimmunized pigs developed high fever, respiratory symptoms, and ataxia at 2 dpc and died successively beginning at 4 dpc (Figures. 6D and E). During the challenge, the piglets were weighed daily, the body weight ratio of the piglets in all the groups tended to increase, and that of the piglets in the rPRV-VP2 vaccine group was the highest (Figure 6F). All the unimmunized pigs died within 10 days, whereas the pigs immunized with the rPRV-VP2 or PRV vaccine survived (Figure 6G). Viral loads in the serum at different time points and in various tissues from all the piglets were quantified by qPCR. The viral load was detected only in the serum of unimmunized piglets, with a rapid increase peaking at 3 dpc, followed by a gradual decline. No viral load was detected at any time point in the serum of immunized piglets (Figure 6H). qPCR analysis revealed that the viral load was detected in all tissues of unimmunized piglets, with the highest levels detected in the tonsils (2.8 × 10⁶ copies/mg). In contrast, the vaccinated groups presented a lower and more limited viral distribution (Figure 6I).

### Anatomical and histopathological observations of pigs after PRV challenge

**Figure 7:**
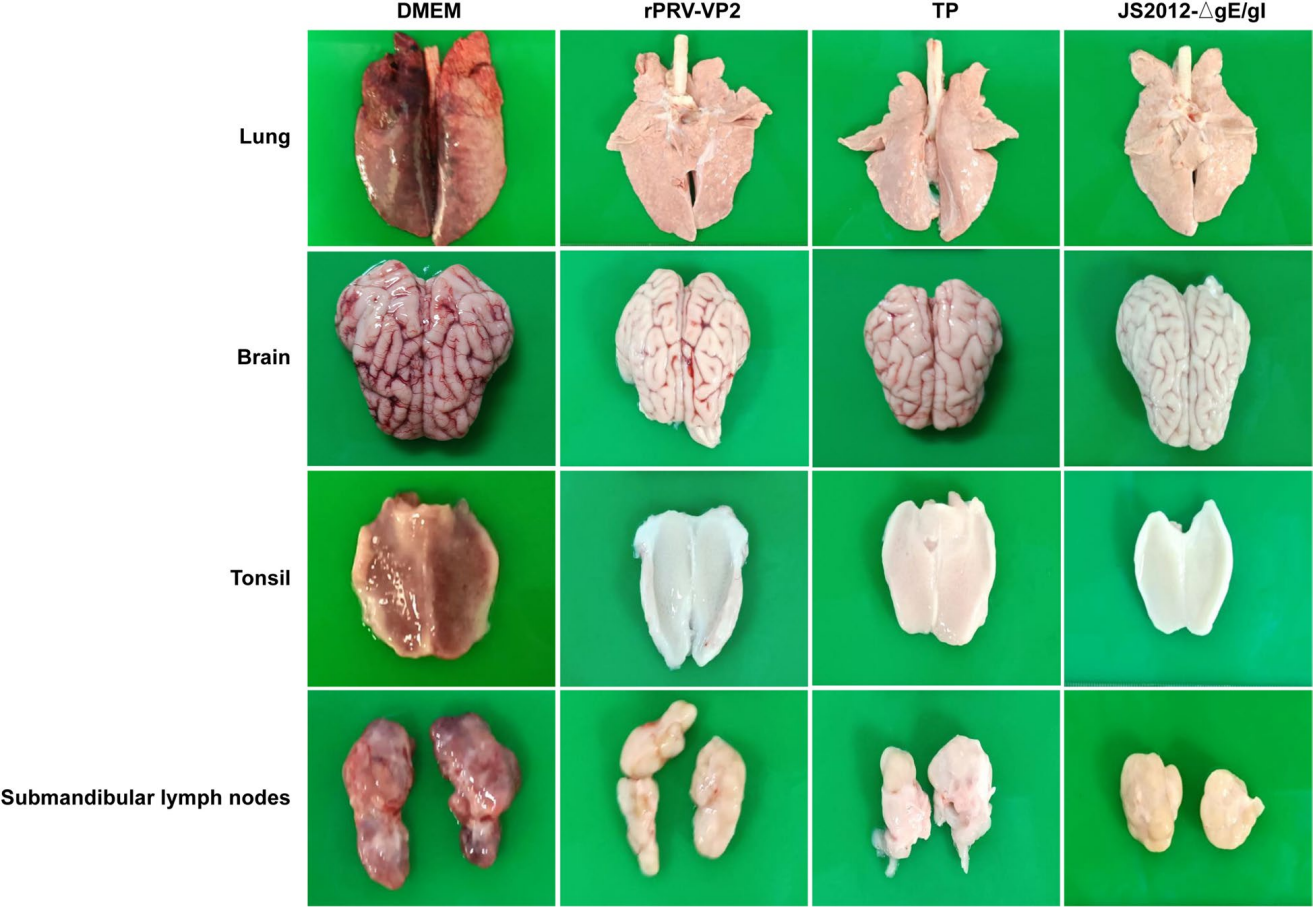
**Observation of pathological changes in piglet tissues after PRV challenge.** Piglets in the DMEM control group exhibited extensive haemorrhage and congestion in the lungs, brain, tonsils, and submandibular lymph nodes. In contrast, those in the immunized group presented intact organ structures with normal colour and texture and no pathological alterations.

**Figure 8:**
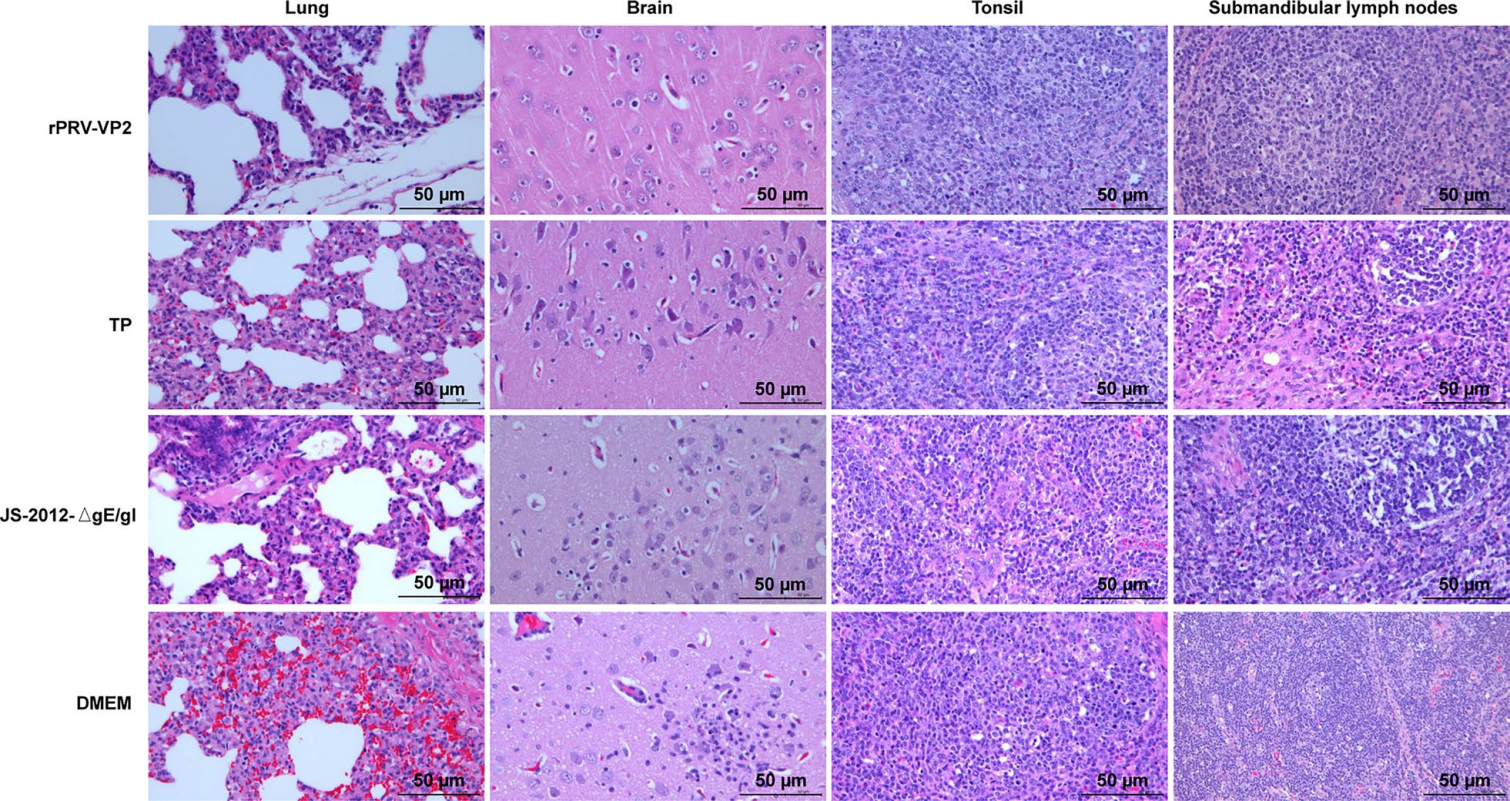
**Histopathological changes in piglet tissues after PRV challenge.** The bar representing the histological images of the lesions represents 50 μm.

Tonsil, brain, lung, and submandibular lymph node tissues were collected from piglets for anatomical and histopathological observation. The results revealed that the control group exhibited extensive haemorrhage and congestion in the tonsils, brain, lungs, and lymph nodes, whereas the piglets in the immunized group presented intact organ structures with a normal colour and texture and no pathological changes (Figure 7). Histopathological examination revealed neuronal cell necrosis in the brain, inflammatory cell infiltration and local haemorrhage in the lungs, as well as alveolar epithelial cell proliferation and lymphocyte necrosis in the control group. While some minor pathological changes were observed in certain tissues of the experimental group, the overall lesions were less severe, which may be related to the fact that the pigs used in this experiment were sourced from farms rather than SPF pigs (Figure 8).

### Protection of piglets vaccinated with rPRV-VP2 against PPV1 challenge

**Figure 9:**
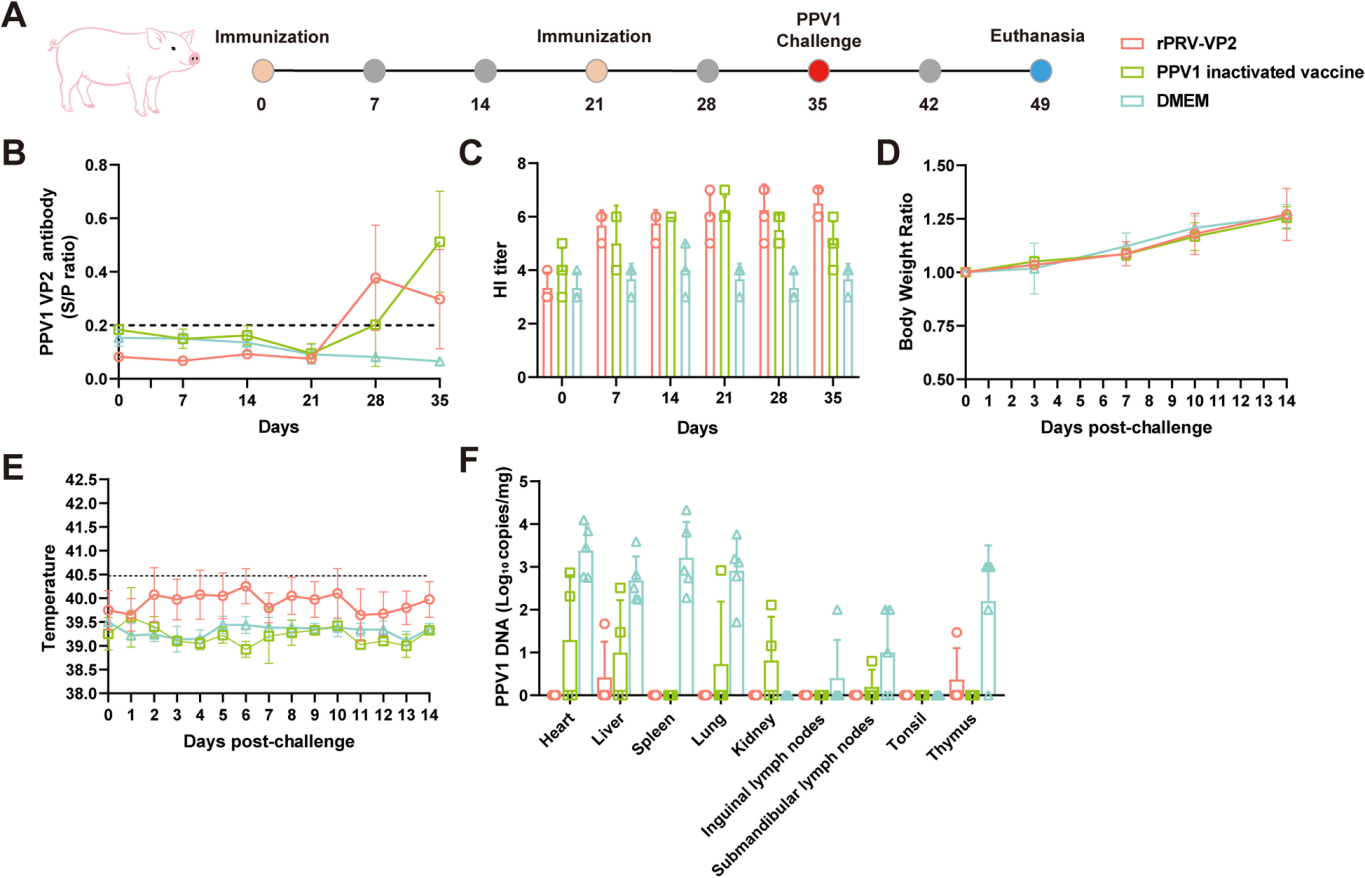
**Protection of pigs vaccinated with rPRV-VP2 against PPV1 challenge. A** Schematic of the rPRV-VP2 immunization procedure in pigs. **B** PPV1 VP2-specific Ab levels in pigs immunized with rPRV-VP2. **C** HI antibody levels in pig sera after immunization. The daily weight gain rate (**D**) and rectal temperature (**E**) were monitored for the indicated times. **F** Viral load in tissues detected by qPCR.

The immunogenicity of the rPRV-VP2 recombinant virus was compared with that of the PPV1 inactivated vaccine. After the initial immunization, a booster dose was administered at 21 dpi, followed by viral challenge two weeks later (Figure 9A). At 28 dpi, pigs immunized with rPRV-VP2 and the inactivated PPV1 vaccine had seroconverted for VP2-specific antibodies (Figure 9B). In the rPRV-VP2-immunized group, HI titres continued to rise from the first immunization through the challenge. In contrast, the HI titre in the PPV1 inactivated vaccine group peaked at 21 dpi and gradually declined thereafter (Figure 9C). During the challenge, piglet body weight and temperature were measured daily, and the piglets maintained a normal body temperature and stable weight gain (Figures. 9D and E). Moreover, PPV1 was detected in the heart, liver, spleen, lungs, thymus, submandibular, and inguinal lymph nodes of the unimmunized pigs, with the highest viral load observed in the heart tissue at 2.4 × 10^3^ copies/mg. In contrast, the virus was also detectable in some tissues of the inactivated vaccine- and rPRV-VP2-immunized groups, but the viral load was lower than that in the nonimmunized group (Figure 9F).

## Discussion

Vaccination is one of the most effective prophylactic strategies for protecting pigs against viral infections. In intensive pig production systems, simultaneous infection with multiple pathogens is common [35]. Therefore, bivalent or multivalent vaccines play a key role in protecting against multiple diseases and simplifying immunization procedures. The large genome of PRV contains many nonessential regions, such as TK, gE, gI, and gG, which can accommodate several kilobases of foreign DNA. Thus, PRV has evolved into an effective vector for expressing foreign proteins, such as CSFV E2 protein, PCV2 Cap protein, and PPV1 VP2 protein. Most rPRV can induce the production of specific antibodies in animals and provide complete protection. The protective efficacy of a rPRV expressing the PPV1 VP2 protein has been previously investigated in mice and pigs [23, 24]. However, the immune efficacy was not satisfactory in vivo. In recent years, various strains of PPV1 have been reported [13]. To enhance protective efficacy, all available complete PPV1 VP2 gene sequences (*n* = 143) in China were downloaded from GenBank, and a consensus sequence was generated. The resulting sequence was subsequently inserted into the PRV genome to construct a recombinant PRV strain, designated rPRV-VP2, which successfully expresses the PPV1 VP2 protein. We then evaluated the efficacy of this strain as a candidate recombinant marker vaccine against challenges with virulent PRV and PPV1 strains. First, we constructed PRV-ΔUL39/TK/gE/gI, a gene-deleted strain with UL39, TK, gE, and gI deletions, on the basis of the currently circulating PRV variant strain (HLJ8) in China. The safety and immunogenicity of PRV-ΔUL39/TK/gE/gI were subsequently evaluated in pigs. The results showed that the virus was safe and immunogenic for pigs, suggesting that PRV-ΔUL39/TK/gE/gI could serve as a biologically safe vaccine vector for expressing foreign proteins. To verify this, the PPV1 VP2 expression cassette was inserted into the region of the gE and gI genes to generate the recombinant rPRV-VP2, which remained genetically stable with no mutations in the VP2 expression cassette during in vitro passage. Compared with parental PRV HLJ8, the insertion of the VP2 expression cassette had little effect on the in vitro replication of rPRV-VP2. These findings suggest that the region of the gE and gI genes is an excellent site for accommodating the insertion of foreign genes. Moreover, rPRV-VP2 exhibits hemagglutination activity with a hemagglutination titre of 2^6^ (1:64), indicating that the VP2 protein correctly displays the epitope of PPV1 required for hemagglutination activity and that the structure is similar to that of the native PPV VP2 protein.

The immunoprotective efficacy of rPRV-VP2 against PRV was initially evaluated in mice. The mice showed seroconversion of PRV gB-specific antibodies at 14 dpi. Similarly, VP2-specific antibodies seroconverted at 14 dpi and remained at high levels at 28 and 35 dpi. Throughout the entire immunization period, no significant clinical symptoms were observed in the mice. Moreover, the mice immunized with rPRV-VP2 were resistant to PRV HLJ8 infection, whereas the unimmunized mice exhibited itching and bite behaviour, with 83.33% (5/6) of the mice succumbing to the infection. These results indicate that the rPRV-VP2 vaccine candidate is safe for mice and effectively induces the production of specific antibodies, providing complete protection against PRV HLJ8 infection. The immune protective efficacy of rPRV-VP2 against PRV was subsequently evaluated in piglets and compared with that of commercial PRV vaccines. PRV gB-specific antibodies were detected in all immunized pigs at 7 dpi, and there were no significant differences between pigs immunized with the rPRV-VP2 vaccine and those immunized with the inactivated PRV vaccine. All pigs were challenged with PRV HLJ8 at 35 dpi, and all the unimmunized pigs died within 10 days. Piglets in the PRV inactivated vaccine group commonly exhibited clinical symptoms such as fever and respiratory distress, with some individuals developing severe diarrhoea and approaching a moribund state. In contrast, no significant clinical symptoms were observed in the commercial live attenuated vaccine and rPRV-VP2 immunization groups. The amount of viral nucleic acid in various tissues of the immunized pigs was significantly lower than that in the unimmunized pigs. Furthermore, the immunoprotective efficacy of rPRV-VP2 against PPV1 was compared with that of a commercial PPV1 inactivated vaccine. Compared with pigs immunized with the inactivated PPV1 vaccine, pigs immunized with rPRV-VP2 seroconverted at 28 dpi, with no significant differences observed. Additionally, hemagglutination inhibition titres were significantly greater than those in the DMEM-immunized group, but no significant difference was noted compared with those in the PPV1 vaccine-immunized group. As PPV1 primarily affects sow reproduction, the piglets in all the experimental groups presented no apparent clinical symptoms. However, the viral load in the tissues of piglets in the control group was significantly greater than that in the immunized group, indicating that the antibodies produced by the immunized piglets were capable of effectively neutralizing the virus.

Over the past three decades, PRV has been extensively utilized as a vector for expressing foreign proteins. However, no commercially available PRV vector vaccine has been developed to date. While constructing PRV vector vaccines is no longer technically challenging, issues such as the loss of inserted foreign genes remain prevalent. Moreover, some studies have only assessed the immunogenicity of PRV vector vaccines in mice without further evaluating their immune efficacy in piglets, which has hindered the commercialization of these vaccines. Maternal antibody interference remains a key obstacle to the application of rPRV; however, recent progress in needle-free intradermal immunization has provided an effective means to overcome this challenge. Notably, the recombinant virus rDEVus78Ha, which uses duck plague virus as a vector to express the hemagglutinin (HA) gene of the H5 subtype avian influenza virus, has been officially approved and commercially launched [36]. Since both PRV and duck plague virus belong to the alpha herpesvirus subfamily, the construction of recombinant viruses expressing foreign proteins via the use of PRV as a vector holds great potential for future applications.

In this study, we generated the consensus sequence of the PPV1 VP2 gene and inserted it into the PRV genome. The recombinant virus was passaged for 20 generations, with the foreign gene remaining stable. We evaluated the immune efficacy of the vaccine in both mice and piglets, confirming its ability to induce the production of specific antibodies and provide complete protection. These findings provide a promising strategy for the development of combined vaccines against PRV and PPV1, offering a new approach for improving disease prevention in swine.

## Supplementary Information

**Additional file 1. SgRNA and primers used in this study.**

**Additional file 2. Recombination and purification of rPRV-eGFP. **(A) Schematic diagram of the rPRV-eGFP construction strategy. (B) Recombination of rPRV-eGFP. The PRV-△UL39/TK virus genome, pX330-sgRNA gE, linearized pCA-arm-eGFP plasmid, and pX330-sgRNA gI were transfected into BHK21 cells, and fluorescent lesions were observed at 30 h. (C) Plaque purification of rPRV-eGFP. rPRV-eGFP fluorescence was obtained after six rounds of plaque purification.

**Additional file 3. Comparison of sgRNA knockout efficiency for the eGFP gene.** (a) Cells transfected with the pCAGGS-eGFP plasmid alone; cells cotransfected with the pCAGGS-eGFP plasmid and (b) pX330-sgRNA eGFP 1; (c) pX330-sgRNA eGFP 2; (d) pX330-sgRNA eGFP 3; (e) pX330-sgRNA eGFP 4; (f) pX330-sgRNA eGFP 5; (g) pX330-sgRNA eGFP 6; (h) pX330-sgRNA eGFP 7; (i) control.

## Data Availability

The data that support the findings of this study are available from the authors upon reasonable request.
