# Supplementary material for: Recombinant pseudorabies virus expressing the consensus VP2 protein of porcine parvovirus 1 (PPV1) protects pigs against pseudorabies virus and PPV1
Source: Vet Res. 2025 Aug 5;56:162. doi: 10.1186/s13567-025-01592-y (PMC12326671; doi:10.1186/s13567-025-01592-y)
Supplement: Supplementary file 1 — Additional file 1. SgRNA and primers used in this study. [file 13567_2025_1592_MOESM1_ESM.docx]

Additional file 1. sgRNA and primer used in this study

| Name | Sequence |
| --- | --- |
| pX330-sgRNA TK1 | CACCcatcagcgcggcggccttcg |
|  | AAACcgaaggccgccgcgctgatg |
| pX330-sgRNA TK2 | CACCggaggtgacggagtccgcgta |
|  | AAACtacgcggactccgtcacctcc |
| pX330-sgRNA gE | CACCcgctccggcttcgacgtctgg |
|  | AAACccagacgtcgaagccggagcg |
| pX330-sgRNA gI | CACCtggtggcgcgcgacgtgacc |
|  | AAACggtcacgtcgcgcgccacca |
| pX330-sgRNA eGFP 1 | CACCcaactacaagacccgcgccg |
|  | AAACcggcgcgggtcttgtagttg |
| pX330-sgRNA eGFP 2 | CACCatcggcgacggccccgtgct |
|  | AAACagcacggggccgtcgccgat |
| pX330-sgRNA eGFP 3 | CACCaccgccgccgggatcactct |
|  | AAACagagtgatcccggcggcggt |
| pX330-sgRNA eGFP 4 | CACCgagctggacggcgacgtaaa |
|  | AAACtttacgtcgccgtccagctc |
| pX330-sgRNA eGFP 5 | CACCaacgagaagcgcgatcacat |
|  | AAACatgtgatcgcgcttctcgtt |
| pX330-sgRNA eGFP 6 | CACCgcgccgaggtgaagttcgag |
|  | AAACctcgaacttcacctcggcgc |
| pX330-sgRNA eGFP 7 | CACCcaacatcgaggacggcagcg |
|  | AAACcgctgccgtcctcgatgttg |
| UL39-F | tcacccccatcgtctctc |
| UL39-R | cgtgcagcgaggtcaggt |
| gE/gI-F1 | tggcctggtaccgcatcg |
| gE/gI-R1 | caaacgtgtccatgtcga |
| gE/gI-F2 | cccaacctgacgatagac |
| gE/gI-R2 | tacaccggagagagcatg |
| TK-F1 | gtagaagcggttgtggcag |
| TK-R1 | cgggcacggcaaactttat |
| TK-F2 | gggcccgcccccagccgca |
| TK-R2 | acccccatctccgacgtgaa |
| pCA eGFP-F | CATCATTTTGGCAAAatggtgagcaagggcgag |
| eGFP-R | TTACTTGTACAGCTCGTC |
| Left arm-F | AAAGTGCCACCTGGGggaccggctgctgaacga |
| Left arm-R | AATAATCAATGTCGAcatcgacgccggtactgc |
| Right arm-F | GACGAGCTGTACAAGTAAaaccggtccgcccgcatt |
| Right arm-R | AAAAAGATCTGCTAGggcccccgagttcaggta |
| VP2-F | CACAACAGAAGGGGACCA |
| VP2-R | GCTCTTCCGCACTTTGAC |
