# Supplementary figures and images for: Recombinant pseudorabies virus expressing the consensus VP2 protein of porcine parvovirus 1 (PPV1) protects pigs against pseudorabies virus and PPV1
Source: Vet Res. 2025 Aug 5;56:162. doi: 10.1186/s13567-025-01592-y (PMC12326671; doi:10.1186/s13567-025-01592-y)

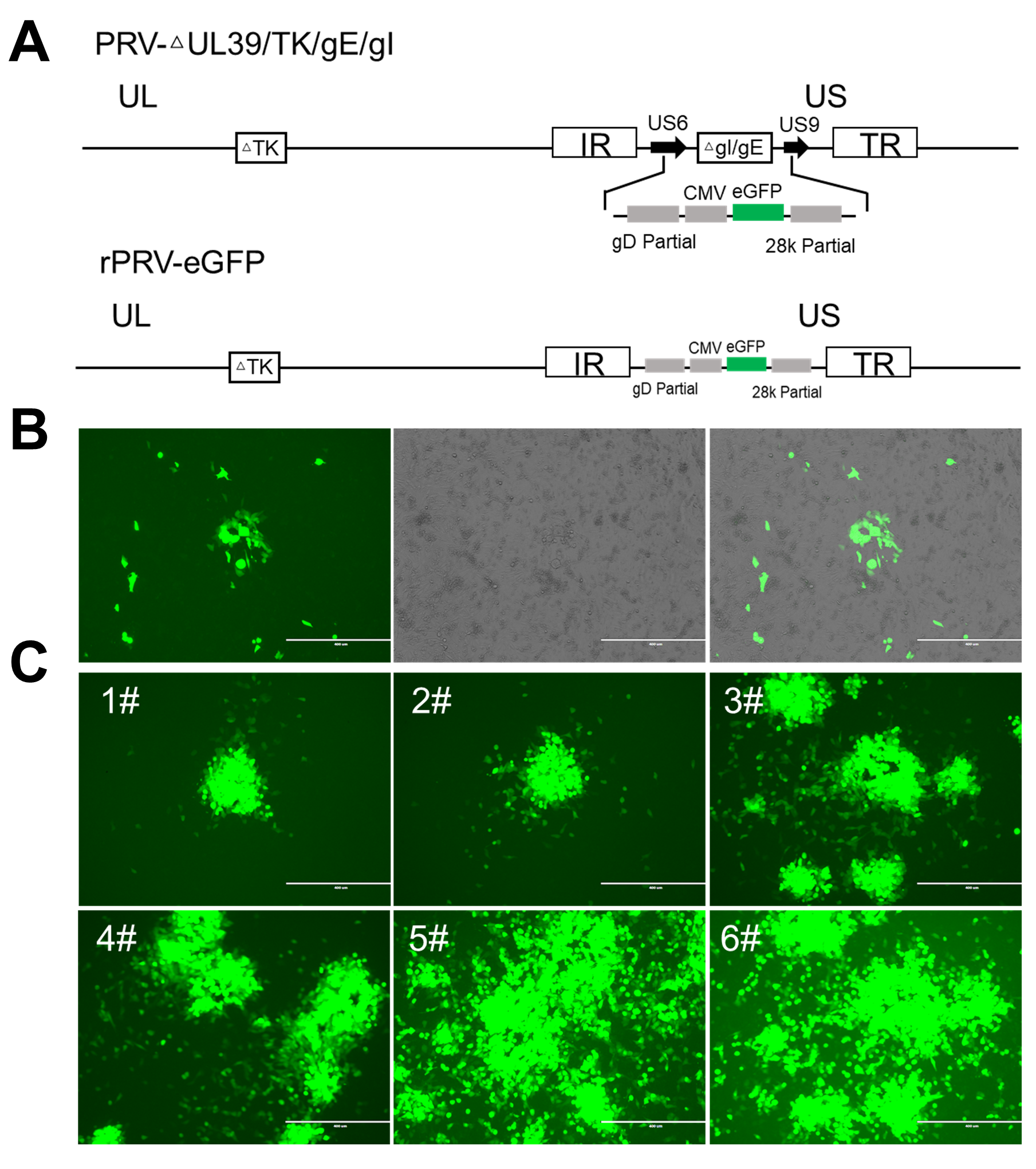

Supplement: Supplementary file 2 — Additional file 2. Recombination and purification of rPRV-eGFP. (A) Schematic diagram of the rPRV-eGFP construction strategy. (B) Recombination of rPRV-eGFP. The PRV-△UL39/TK virus genome, pX330-sgRNA gE, linearized pCA-arm-eGFP plasmid, and pX330-sgRNA gI were transfected into BHK21 cells, and fluorescent lesions were observed at 30 h. (C) Plaque purification of rPRV-eGFP. rPRV-eGFP fluorescence was obtained after six rounds of plaque purification. [file 13567_2025_1592_MOESM2_ESM.tif]

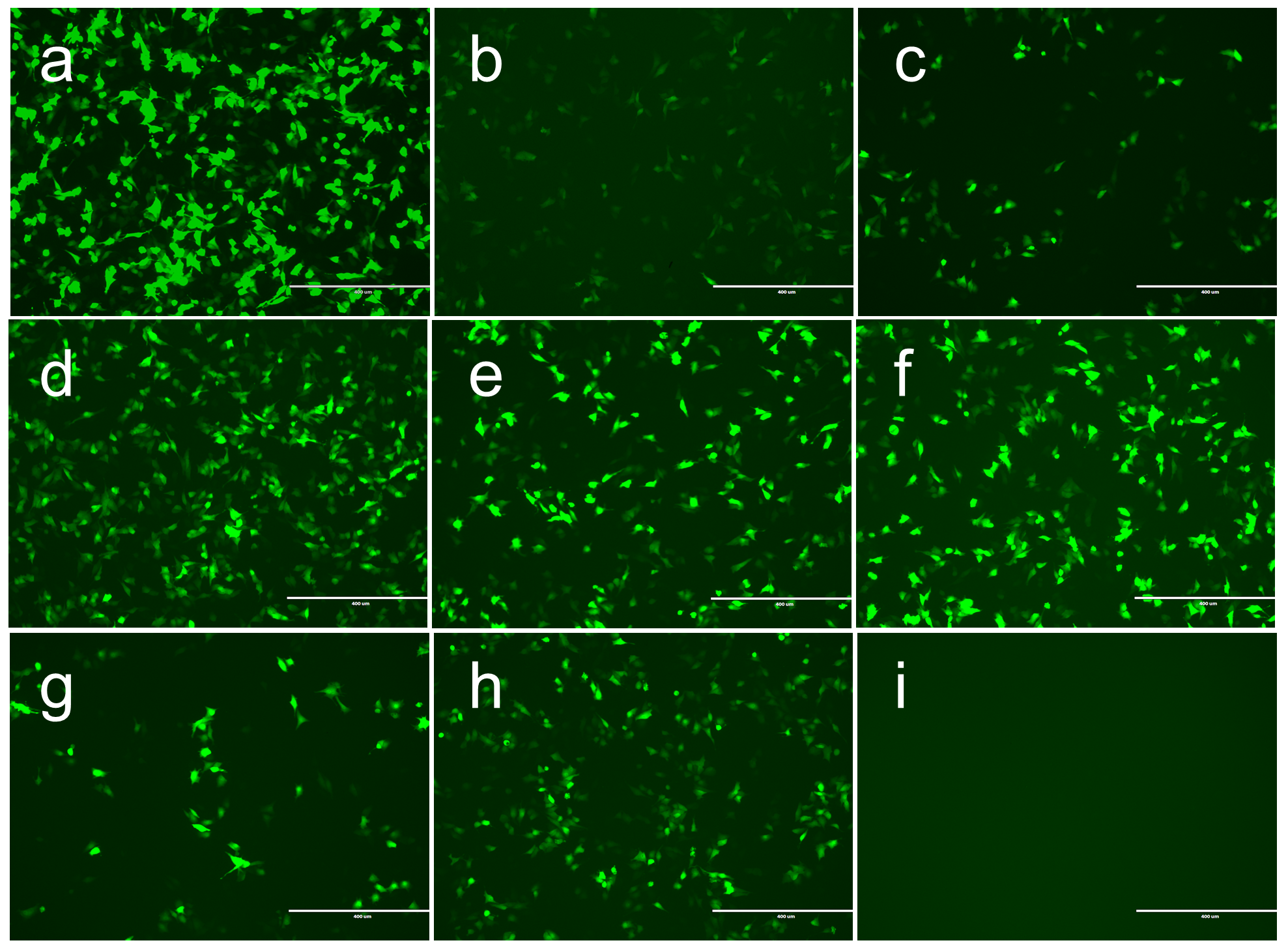

Supplement: Supplementary file 3 — Additional file 3. Comparison of sgRNA knockout efficiency for the eGFP gene. (a) Cells transfected with the pCAGGS-eGFP plasmid alone; cells cotransfected with the pCAGGS-eGFP plasmid and (b) pX330-sgRNA eGFP 1; (c) pX330-sgRNA eGFP 2; (d) pX330-sgRNA eGFP 3; (e) pX330-sgRNA eGFP 4; (f) pX330-sgRNA eGFP 5; (g) pX330-sgRNA eGFP 6; (h) pX330-sgRNA eGFP 7; (i) control. [file 13567_2025_1592_MOESM3_ESM.tif]
